# Three new species of *Primulina* (Gesneriaceae) from limestone karsts of China based on morphological and molecular evidence

**DOI:** 10.1186/s40529-015-0115-5

**Published:** 2015-12-12

**Authors:** Jing Guo, Bo Pan, Jing Liu, Wei-Bin Xu, Kuo-Fang Chung

**Affiliations:** 1 College of Life Sciences, Guangxi Normal University, Guilin, 541004 China; 2 Guangxi Key Laboratory of Plant Conservation and Restoration Ecology in Karst Terrain, Guangxi Institute of Botany, Guangxi Zhuang Autonomous Region and Chinese Academy of Sciences, Guilin, 541006 China; 3 School of Forestry and Resource Conservation, National Taiwan University, Taipei, 106 Taiwan

**Keywords:** Flora of Guangdong, Molecular taxonomy, *Primulina maculata*, *Primulina pengii*, *Primulina yangshanensis*, Sino-Vietnamese limestone karst

## Abstract

**Background:**

With more than 160 described species, *Primulina* is one of the most characteristic plant groups of the Sino-Vietnamese limestone flora. In our continous botanical inventory of the limestone flora of South China, we collected three new *Primulina* species not identifiable to known species.

**Results:**

Molecular phylogenetic analyses based on nuclear ITS and chloroplast *trnL*-*F* and *trnH*-*psbA* sequences strongly support the placement the three new species in *Primulina*. In addition to morphological differences, DNA sequences of all these three new species show substantial divergencies, sustaining the recognition of these three new species.

**Conclusions:**

Based on morphological and molecular data, we describe and illustrate three new *Primulina* species: *P. maculata*, *P. pengii*, and *P. yangshanensis*.

## Background

As a consequence of recent molecular recircumscription (Wang et al. 2011; Weber et al. 2011), *Primulina* Hance has been expanded greatly from a monotypic genus (Wang et al. 1990; Ying et al. 1993; Wang et al. 1998; Li and Wang 2004; Zheng and Xia 2005; Wei 2010) to one of the largest genera of the Old World Gesneriaceae (Xu et al. 2013). In the past few years, a soaring number of new *Primulina* species are being described (e.g., Huang et al. 2012; Wu et al. 2012; Xu et al. 2012a; Chung et al. 2013; Pan et al. 2013; Xu et al. 2013; Zhao et al. 2013; Li et al. 2014; Zheng and Deng 2014; Li et al. 2015; Ning et al. 2015). Currently, *Primulina* comprises ca. 160 predominately calciphilous species distributed almost exclusively to limestone karsts of China south of Yangtze River and northern Vietnam (Xu et al. 2012a, b; Chung et al. 2013; Xu et al. 2013; Li et al. 2015). Among them, ca. 130 species (85 %) are described from southern and southwestern China (i.e., Guangxi, Guangdong, and Yunnan), an area recognized as a global biodiversity hotspot (Xu 1995; Hou et al. 2010; López-Pujol et al. 2011), with a majority of the species known only from a single or a handful of localities (Xu et al. 2012a; Chung et al. 2013; Xu et al. 2013; Chung et al. 2014).

However, because *Primulina* was circumscribed based on molecular evidence and lacks apparent synapomorphies (Weber et al. 2011), some species might have been misplaced (e.g., Christie et al. 2012; Xu et al. 2014). Moreover, because many of the newly described species are differed only by trivial morphological differences, Weber et al. (2011) suspects that the diversity in *Primulina* is likely overestimated. Therefore, it is essential to combine molecular data with morphological observation for generic placement and species description, taking into account of both morphological and genetic divergences (e.g., Xu et al. 2012a; Chung et al. 2013; Pan et al. 2013; Xu et al. 2013).

During our botanical explorations in limestone karsts of South China, we discovered three distinct species of *Primulina* not identifiable to any known species. This article provides molecular and morphological evidence for the description of these three new species.

## Methods

### Phylogenetic analyses

Total genomic DNA of the three new species was extracted from silica-gel dried leaves collected from the type locality using the CTAB protocol (Xu et al. 2012a). DNA sequences of the nuclear internal transcribed spacers (ITS) and the chloroplast intergenic spacer *trnL*-*F*, which were used for the recircumscription of *Primulina* (Wang et al. 2011; Weber et al. 2011), were amplified and sequenced based on the PCR procedures outlined in Xu et al. (2012a). Additionally we also sequenced chloroplast *trnH*-*psbA* sequences that are amply available in the GenBank based on PCR conditions detailed in Kang et al. (2014).

In our initial BLAST search through GenBank using DNA sequences of the three new species, the closest relatives with the highest hit scores were species in the recently recircumscribed *Primulina* as expected based on morphological observations. To further elucidate phylogenetic affinities of these new taxa, we included sequences of 92 species for ITS, 58 species for *trnL*-*F*, and 53 species for *trnH*-*psbA* of *Primulina* available in GenBank. The species and the GenBank accession numbers are listed in Appendix 1. *Petrocodon dealbatus* Hance, *Petrocodon scopulorus* (Chun) Yin Z. Wang, and *Petrocodon guangxiensis* (Yan Liu & W.B. Xu) W.B. Xu & K.F. Chung were chosen as outgroups based on recent phylogenetic analyses (Xu et al. 2014). The final analyses includes 218 DNA sequences of the three DNA regions in 101 species with 15 newly obtained. Sequences were aligned using MUSCLE 3.8.31 (Edgar 2004) and adjusted manually in Bioedit 5.0.9 (Hall 1990).

The aligned sequences were first analyzed separately using maximum likelihood (ML) and Bayesian inference (BI) approaches. Optimal substitution models for each data set were determined using the Akaike information criterion (AIC) using jModeltest 0.1.1 (Posada 2008) with GTR + Γ selected as the best model. ML analyses of each datasets were performed using RAxML 7.0.3 (Stamatakis et al. 2008), with the GTR + Γ model of sequence evolution selected and the option of a rapid bootstrap analyses (1000 replicates) and a search for the best-scoring tree in a single program run. Bayesian analyses were executed in MrBayes 3.1.2 (Ronquist and Huelsenbeck 2003), using the GTR + Γ model for each datasets. Bayesian analyses were started from random trees, sampling one tree every 1000th generation, with four incrementally heated chains. The Markov chain Monte Carlo (MCMC) algorithm was run with two replicates for 400,000,000 generations for each data set. Analyses were run until the average standard deviation of the split frequencies approached 0.01, indicating that two runs converged to a stationary distribution. The first 5000 trees corresponding to the ‘burn-in’ period were discarded, and the remaining trees were used to construct a majority-rule consensus tree. Posterior probability (PP) was used to estimate robustness. We used the parsimony-based incongruence length difference (ILD) test (Farris et al. 1994) to test for phylogenetic incongruence between cpDNA and nrDNA data sets. Although the ILD test indicated that the two datasets are incongruent (P = 0.01), in the combined analyses framework, the interaction of characters from the two incongruent partitions actually increase phylogenetic resolution and support than that observed in separate analyses of individual data sets (DeSalle et al. 2002). Consequently these three DNA sequence regions were combined into a concatenated matrix and analyzed using ML and BI methods with same parameters stated above.

## Results

### Phylogenetic analyses

**Fig. 1:**
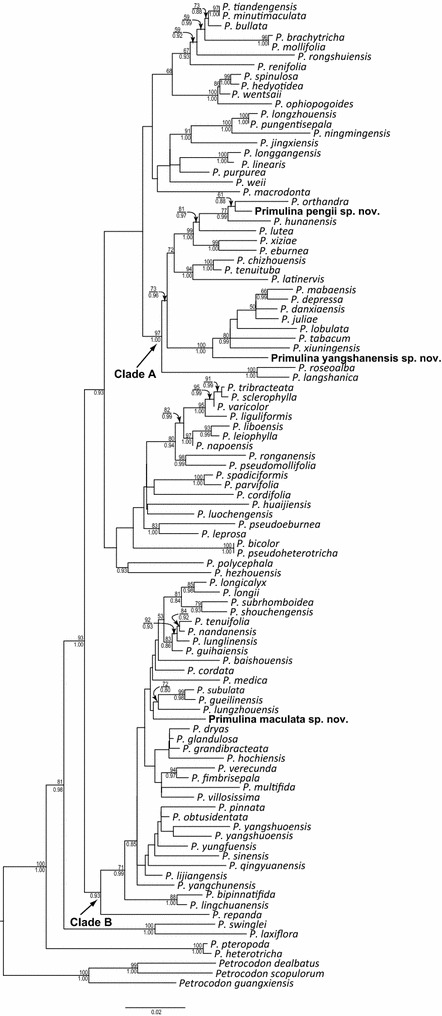
The best ML phylogram based on the combined molecular data set. ML bootstrap support >50 % and Bayesian posterior probability >0.80 are indicated on and below the branches, respectively. The three new species are highlighted in bold

The aligned positions of ITS, *trnL*-*F* and *trnH*-*psbA* datasets were 722, 852 and 643 base pairs, respectively. The combined matrix of the three markers consisted of 2219 characters. The best ML phylogram, with bootstrap (BS) supports and PP values of Bayesian analyses, are depicted in Fig. 1.

Phylogenetic relationships of the concatenated matrix are congruent with those reported in Kang et al. (2014) and support the placement of the three new species (*Primulina maculata*, *P. pengii*, and *P. yangshanensis*) in *Primulina* (BS = 100 %; PP = 1.00). Additionally DNA sequences of all these three species show substantial differences from other species of *Primulina* (Fig. 1), ascertaining their recognition as distinct species. Within *Primulina*, *P. maculata* was placed in Clade B, allied with *P. gueilinensis*, *P. subulata*, and *P. lungzhouensis* with no support (Fig. 1). Interestingly, none of these latter three species have the white maculation along the veins. *Primulina pengii*, together with *P. orthandra* and *P. hunanensis*, formed a well-supported clade (BS = 77 %, PP = 0.99) closely allied with *P. lutea*, *P. eburnean*, and *P. xiziae* that all possess big bracts with strong support (BS = 99 %, PP = 1.00). *Primulina yangshanensis* is sister to the group including its morphologically similar species, *P. mabaensis*, with strong support value (BS = 100 %, PP = 1.00).

### Taxonomic treatment

#### ***Primulina maculata*** W.B. Xu & J. Guo, sp. nov. **花葉牛耳朵** (Figs. 2, 3)

TYPE: CHINA. Guangdong Province, Yangchun City, Shiwang Township, alt. 50 m, in the crevice of limestone hills, 16 April 2013, *Wei*-*Bin Xu 11916* (holotype IBK, isotype PE).

**Fig. 2:**
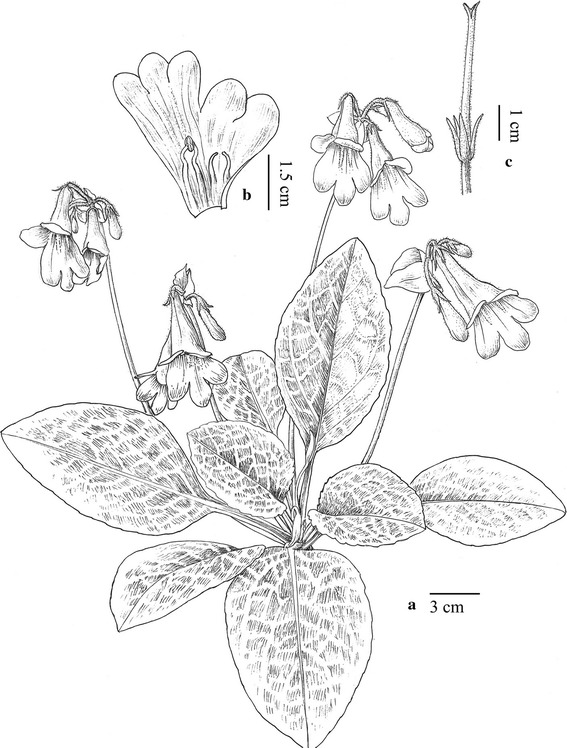
*Primulina maculata* W.B. Xu & J. Guo. **a** Habit; **b** corolla opened showing stamens and staminodes; **c** pistil and calyx (drawn by W. H. Lin from the holotype)

**Fig. 3:**
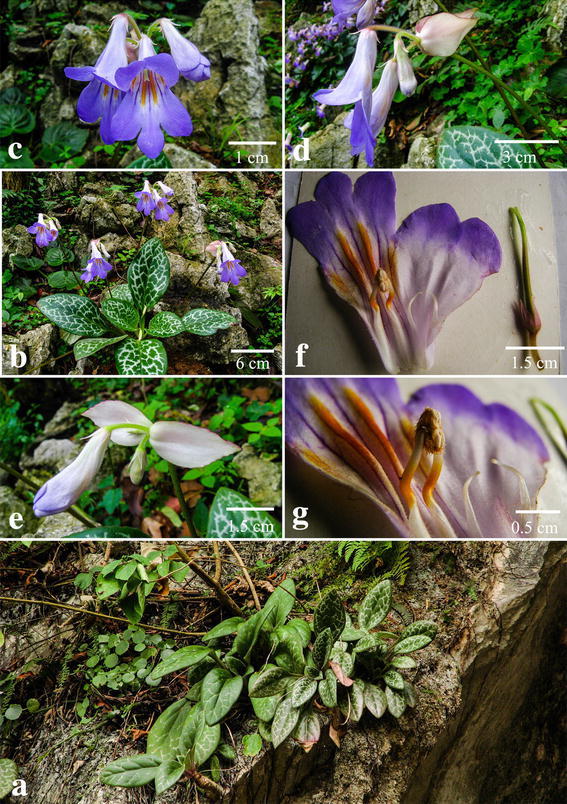
*Primulina maculata* W.B. Xu & J. Guo. **a** Habitat; **b** habit; **c** flower face view; **d** flower side view; **e** bracts; **f** pistil and opened corolla; **g** stamens and staminodes

*Primulina maculata* is most similar to *P. eburnea* (Hance) Y.Z. Wang, a common and widespread congener of South China, but differs in the conspicuous white maculation along the veins (vs. inconspicuous maculation in the latter species).

Herbs perennial rhizome subterete, 4–8 cm long, 1.6–2.2 cm across. Leaves 4–8, crowded at the apex of rhizome, petiolate; petiole applanate, 3–9 cm long, 4–6 mm across; leaf blade fleshy, ovate to elliptic, 5.5–16 × 3.5–7 cm, the apex obtuse, the base cuneate to broadly cuneate, inequilateral, the margin shallowly repand, crenate to entire, pubescent on both surfaces, lateral veins 3–5 on each side, impressed adaxially and prominent abaxially, the white maculation along the veins. Cymes 3–6, 1 or 2-branched, 4–9-flowered; peduncle 26–40 cm long, 2.5–3.5 mm across, densely pubescent; pedicel 1.5–2.8 cm long, densely pubescent; bracts opposite, ovate to lanceolate, 2.8–4.0 × 1.2–1.8 cm, the margin entire to shallowly repand, the apex acuminate, outside densely pubescent, inside sparsely pubescent. Calyx 5-parted nearly to the base, the lobes narrowly lanceolate, 10–12 × ca. 1.5 mm, outside densely pubescent, inside sparsely pubescent. Corolla pale purple, 3.8–5.1 cm long, outside pubescent, inside sparsely puberulent, with 2 pale yellow stripes; corolla tube 2.8–3.5 cm long, 13–17 mm in diam. at the mouth, 4–5 mm in diam. at the base; limb distinctly 2-lipped, pale purple; the adaxial lip 2-parted to over the middle, the lobes broadly ovate, 15–17 × 12–14 mm; the abaxial 3-lobed to over the middle, the lobes oblong, 12–15 × 8–10 mm; stamens 2, adnate to ca. 1.0 cm above the corolla tude base; filaments linear, ca. 1.1 cm long, geniculate over the middle, sparsely puberulent at the base; anthers ca. 5 mm long, ca. 2.5 mm wide, dorsifixed, back densely lanate; staminodes 2, ca. 9 mm long, apex capitate, sparsely puberulent, adnate to ca. 9 mm above the corolla tube base, Disc ringlike, ca. 1.5 mm in height, the margin repand, glabrous. Pistil 2.4–3.5 cm long, ovary 11–18 mm long, ca. 2 mm across, densely puberulent; style 10–16 mm long, ca. 0.7 mm across, puberulent; stigma obtrapeziform, ca. 2 mm long, apex 2-lobed. Capsule not seen.

**Fig. 4:**
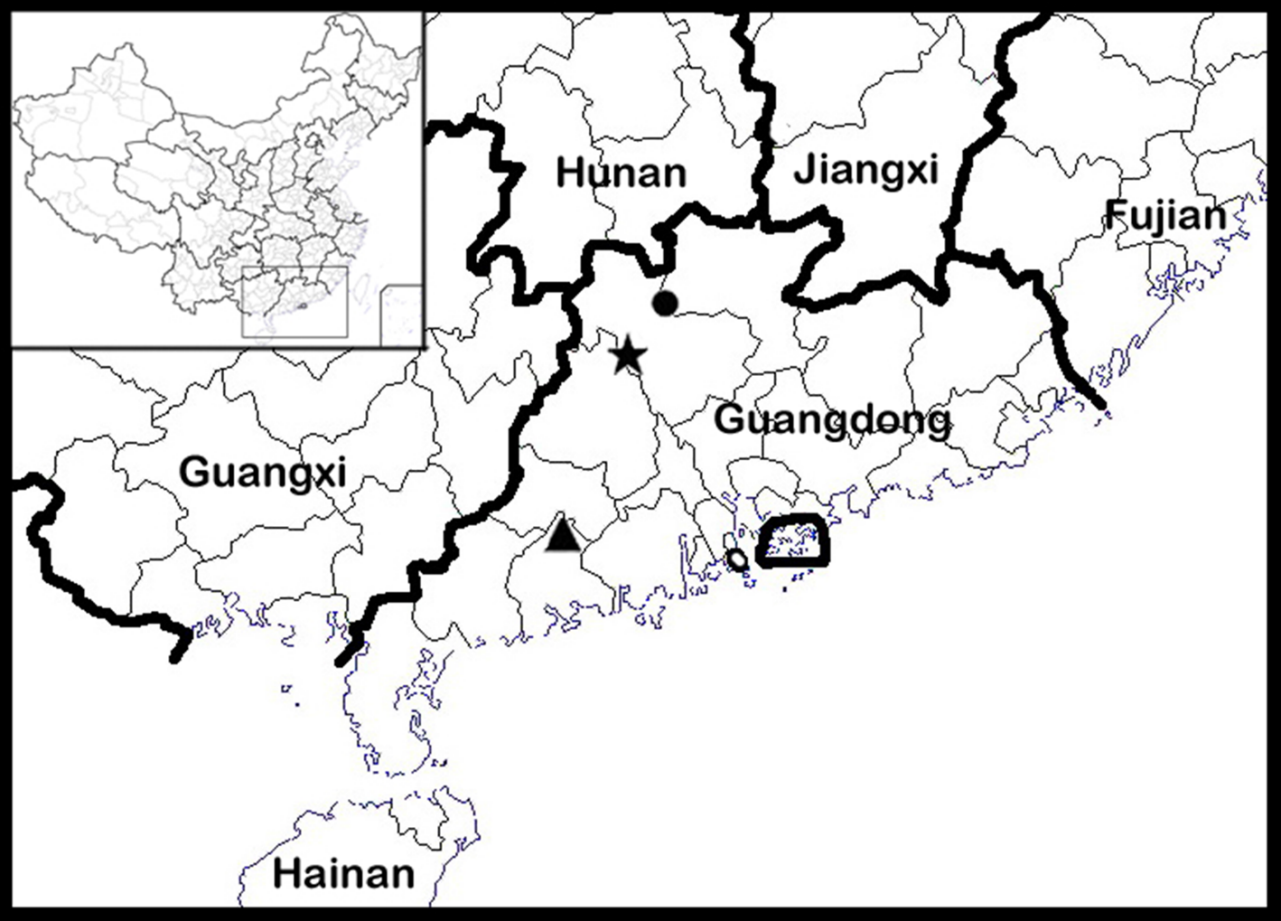
Distribution of *Primulina maculata* (*triangle*), *Primulina pengii* (*circle*), *Primulina yangshanensis* (*star*) in Guangdong Province, China

*Distribution, habitat and ecology Primulina maculata* is known only from the type locality in Shiwang Township, Yangchun City, Guangdong Province, China (Fig. 4). It grows in the crevice of limestone hills. The species flowers from April to May. Fruits have not been observed.

*Etymology* The specific epithet is derived from the white maculation along the veins.

*Additional specimens examined* CHINA. Guangdong Province, Yangchun City, Shiwang Township, 11 April 2014, *Wei*-*Bin Xu & Bo Pan 11949* (IBK); the same locality, 13 August 2012, *Wei*-*Bin Xu* et al*. 11853* (IBK).

*Notes Primulina maculata* is very similar to *P. eburnea* (Hance) Y.Z Wang in overall morphology but can be easily distinguished from the latter by mottled leaves with white maculae along veins. Phylogenetically, these two species are placed in two clades and distantly related (Fig. 1), supporting the recognition of *P. maculata* as a distinct new species.

#### ***Primulina pengii*** W.B. Xu & K.F. Chung, sp. nov. **彭氏報春苣苔** (Figs. 5, 6)

TYPE: CHINA. Guangdong Province, Shaoguan City, Ruyuan County, Luoyang Township, alt. 290 m, on moist rock face in a valley, 28 April 2012, *Wang*-*Hui Wu & Bing*-*Mou Wang W0397* (holotype IBK, isotypes PE, HAST).

**Fig. 5:**
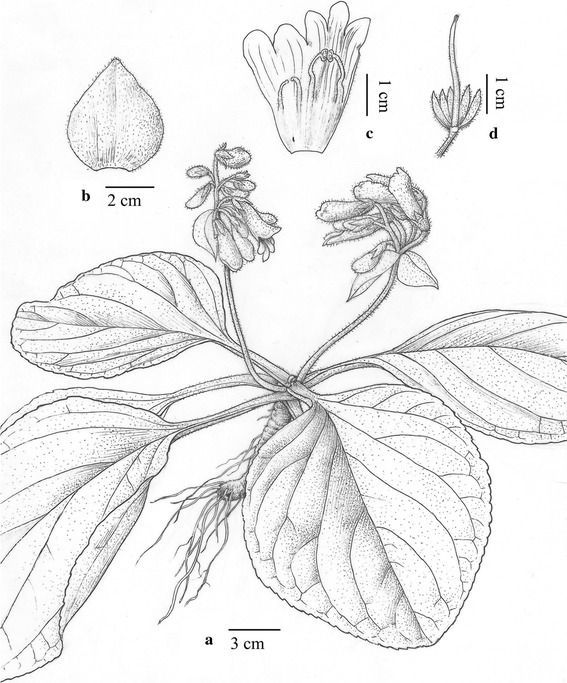
*Primulina pengii* W.B. Xu & K.F. Chung. **a** Habit; **b** bract; **c** corolla opened showing stamens and staminodes; **d** pistil and calyx (drawn by W. H. Lin from the holotype)

**Fig. 6:**
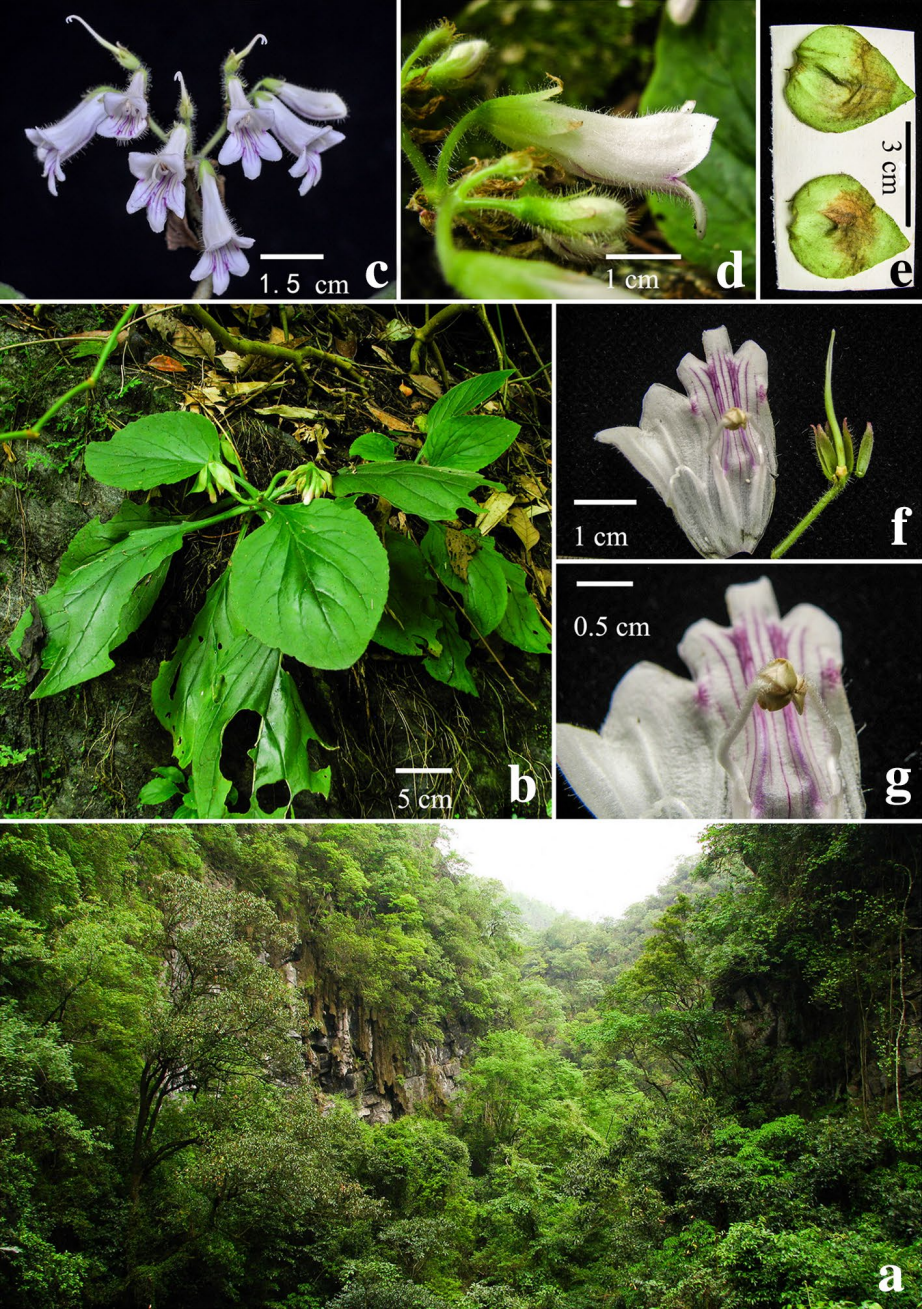
*Primulina pengii* W.B. Xu & K.F. Chung. **a** Habitat; **b** habit; **c** cyme and flower face view; **d** flower side view; **e** bracts; **f** pistil and opened corolla; **g** stamens

*Primulina pengii* is most similar to *P. lungzhouensis* (W.T. Wang) Mich. Möller & A. Weber, differing by the size of leaf blade (14–25 × 9.5–15 vs. 8–18 × 4.5–10 cm), the leaf margin (shallowly repand to crenate vs. dentate or denticulate to serrate), the inflorescence (cymes 1 or 2-branched vs. 1-branched; 4–12-flowered vs. 5–7-flowered), the length of peduncle (5–13 cm long vs. 14–19 cm long), the shape (cordate vs. ovate), size (2.6–3.2 × 2.5–3.0 vs. 2.5–3.8 × 2.2–2.8 cm), and margin (entire to shallowly repand vs. denticulate) of bracts, the color (white vs. pale purple) and length (2.8–3.6 vs. ca. 4.8 cm long) of corolla, and the color of the stripes (pale purple vs. 2 yellow) inside the corolla.

Herbs perennial rhizome subterete, 4–7 cm long, 1.2–1.6 cm across. Leaves 4–6, crowded at the apex of rhizome, petiolate; petiole applanate, 4–9.5 cm long, 4–6 mm across; leaf blade herbaceous, ovate to broadly ovate, 14–25 × 9.5–15 cm, the apex obtuse, the base cuneate to broadly cuneate, inequilateral, the margin shallowly repand to crenate, pubescent on both surfaces, lateral veins 4–6 on each side, impressed adaxially and prominent abaxially. Cymes 3–4, 1–2-branched, 4–12-flowered; peduncle 5–13 cm long, 2.0–3.5 mm across, pubescent; pedicel 6–16 mm long, pubescent; bracts opposite, cordate, 2.6–3.2 × 2.5–3.0 cm, the margin entire to shallowly repand, the apex acute, outside pubescent, inside sparsely pubescent. Calyx 5-parted nearly to the base, the lobes narrowly lanceolate, 8–10 × ca. 2.0 mm, the margin serrulate, outside glandular pubescent, inside sparsely pubescent. Corolla white, 2.8–3.6 cm long, outside glandular pubescent, inside sparsely puberulent, with 2 pale purple stripes; corolla tube 1.9–2.5 cm long, 10–13 mm in diam. at the mouth, 3–4 mm in diam. at the base; limb distinctly 2-lipped, white; the adaxial lip 2-parted to over the middle, the lobes broadly ovate, 5–7 × 6–8 mm; the abaxial 3-lobed to over the middle, the lobes ovate to oblong, 5–7 × 3–4 mm; stamens 2, adnate to ca. 1.5 cm above the corolla tude base; filaments linear, ca. 1.4 cm long, geniculate over the middle, sparsely puberulent; anthers ca. 4 mm long, ca. 2.5 mm wide, dorsifixed, back lanate; staminodes 3, the lateral ones ca. 7 mm long, apex capitate, sparsely puberulent, adnate to ca. 1.2 mm above the corolla tube base, the middle one ca. 1.5 mm, adnate to ca. 3 mm above the corolla tube base. Disc ringlike, ca. 1.5 mm in height, the margin repand, glabrous. Pistil 2.4–3.1 cm long, ovary 15–18 mm long, ca. 1.5 mm across, densely puberulent; style 8–12 mm long, ca. 0.8 mm across, puberulent; stigma obtrapeziform, ca. 1.5 mm long, the apex 2-lobed. Capsule not seen.

*Distribution and ecology Primulina pengii* is known only from the type locality in Luoyang Township, Shaoguan City, Guangdong Province, China (Fig. 4). It grows on moist rock face in a valley. Flowering from April to May, fruiting not observed.

*Etymology* The specific epithet honors Dr. Ching-I Peng of the Herbarium (HAST) of Biodiversity Research Center, Academia Sinica, Taiwan, for his contribution to our knowledge of Sino-Vietnamese karst flora.

*Additional specimens examined (paratypes)* CHINA. Guangdong Province, Shaoguan City, Ruyuan County, Luoyang Township, 19 April 2013, *Ching*-*I Peng* et al*. 24024* (HAST).

*Notes* *Primulina pengii* resembles *P. lungzhouensis*, differing from the latter by the morphology of leaves, bracts, inflorescences, and flowers. Phylogenetically, these two species are placed in two different clades and distantly related (Fig. 1), supporting the recognition of *P. pengii* as a new species.

#### ***Primulina yangshanensis*** W.B. Xu & B. Pan, sp. nov. **陽山報春苣苔** (Figs. 7, 8)

TYPE: CHINA. Guangdong Province, Yangshan County, Qinglian Township, alt. 60 m, in the crevice of limestone hills, 3 May 2012, *Bo Pan & Wei*-*Bin Xu 11697* (holotype IBK).

**Fig. 7:**
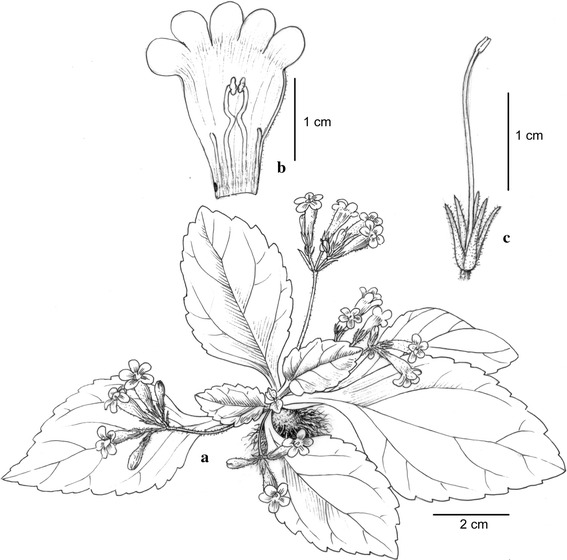
*Primulina yangshanensis* W.B. Xu & B. Pan. **a** Habit; **b** corolla opened showing stamens and staminodes; **c** pistil and calyx (drawn by W. H. Lin from the holotype)

**Fig. 8:**
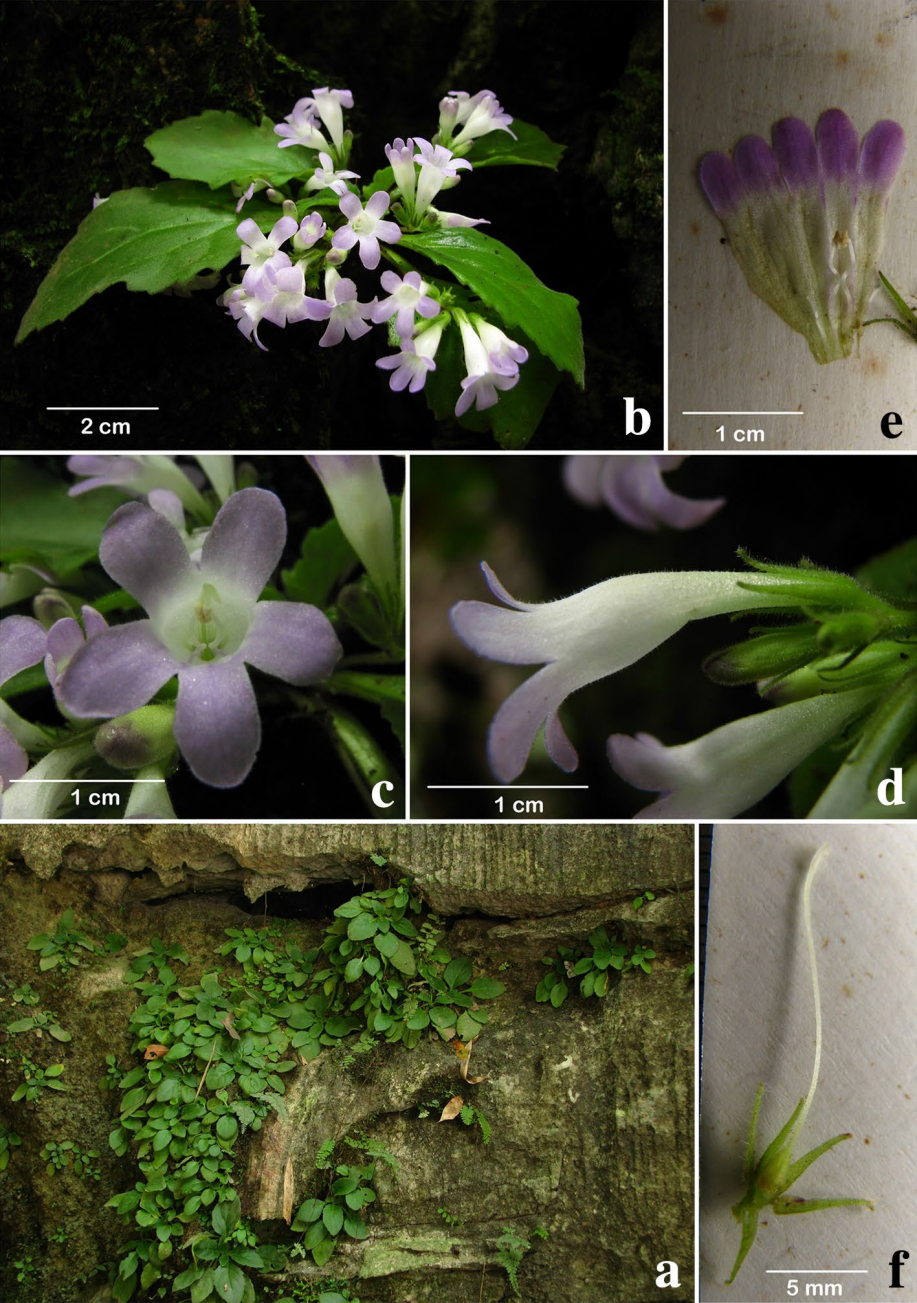
*Primulina yangshanensis* W.B. Xu & B. Pan. **a** Habitat; **b** habit; **c** flower face view; **d** flower side view; **e** opened corolla; **f** pistil and calyx

*Primulina yangshanensis* is most similar to *P. mabaensis* K.F. Chung & W.B. Xu, differing in the shape (ovate to broadly ovate vs. broadly ovate to elliptic) and the apex (acute to obtuse vs. obtuse to rounded) of leaf blade, the number of lateral veins (2 or 3 vs. 3–5 on each side), the length of petiole (0.5–2.5 vs. 3–6 cm), the color of corolla limb (pale purple vs. white), and the curviness of filaments (geniculate near the middle vs. slightly curve).

Herbs perennial. Rhizome subterete, 0.7–2 cm long, 4–7 mm across. Leaves 4–6, crowded at the apex of rhizome; petiole applanate, 0.5–2.5 cm long, 2–3 mm across; leaf blade papery, ovate to broadly ovate, 4.3–7.1 × 2.1–4.3 cm, the apex acute to obtuse, the base cuneate, the margin repand to serrate, pubescent on both surfaces, lateral veins 2–3 on each side, impressed adaxially and prominent abaxially. Cymes 2–5, 1–2-branched, 4–8-flowered; peduncle 3–4.8 cm long, ca. 1 mm across, spreading pubescent; pedicel 3–5.5 mm long, spreading pubescent; bracts opposite, linear-lanceolate, 8–9 × 1.2 mm, the margin entire, the apex acuminate, outside pubescent, inside sparsely pubescent. Calyx 5-parted nearly to the base, the lobes linear-lanceolate, 4–6 × ca. 0.8 mm, the margin entire, outside spreading pubescent, inside sparsely pubescent. Corolla pale purple to white, 1.9–2.6 cm long, outside pubescent, inside sparsely puberulent,; corolla tube white, 1.4–1.7 cm long, 6–8 mm in diam. at the mouth, 3–4 mm in diam. at the base; limb distinctly 2-lipped, pale purple; the adaxial lip 2-parted to near the base, the lobes oblong, 4-5 × 3-4 mm; the abaxial 3-lobed to near the base, the lobes oblong, 5–7 × 3–4 mm; stamens 2, adnate to ca. 5 mm above the corolla tube base; filaments linear, ca. 8 cm long, geniculate near the middle, glabrous; anthers ca. 2 mm long, ca. 1.5 mm wide, dorsifixed, glabrous; staminodes 3, lateral ones ca. 4 mm long, glabrous, adnate to ca. 3 mm above the corolla tube base, middle one ca. 1.5 mm long, adnate to ca. 1.5 mm above the corolla tube base. Disc ringlike, ca. 1 mm in height, the margin repand, glabrous. Pistil 1.8–2.3 cm long, ovary 2–3 mm long, ca. 1.5 mm across, densely puberulent; style 12–17 mm long, ca. 0.5 mm across, puberulent; stigma ca. 1.5 mm long, the apex 2-lobed. Capsule not seen.

*Distribution and ecology Primulina yangshanensis* is known only from the type locality in Qinglian Township, Yangshan County, Guangdong Province, China (Fig. 4). It grows on moist limestone rock face. Flowering from April to May. Fruits not observed.

*Etymology* The specific epithet is derived from the type locality, Yangshan County, Guangdong Province.

*Additional specimens examined* CHINA. Guangdong Province, Yangshan County, Qinglian Township, 10 May 2011, *Bo Pan & Wei*-*Bin Xu 11145 & 11150* (IBK). The same locality, 25 July 2009, *Kuo*-*Fang Chung* et al*. KFC1835* (HAST).

*Notes* *Primulina yangshanensis* is most similar to *P. mabaensis* that is also distributed in the limestone karst of northern Guangdong (Chung et al. 2013). Phylogenetic analyses revealed that these two species are closely related and yet considerably differentiated from one another, supporting the recognition of *P. yangshanensis* as a new species.

## Appendix 1

GenBank accession numbers: Species: (ITS/*trnL*-*F/trnH*-*psbA*)*. Petrocodon dealbatus* Hance: KF498053/KF498254/JX507075; *Petrocodon guangxiensis* (Yan Liu & W.B. Xu) W.B. Xu & K.F. Chung: JX506899/JX506791/JX506995; *Petrocodon scopulorus* (Chun) Y.Z. Wang: HQ633044/GU350669/–; *Primulina baishouensis* (Y.G. Wei, H.Q. Wen & S.H. Zhong) Y.Z. Wang: KF498103/JX506742/JX506962; *Primulina bicolor* (W.T. Wang) Mich. Möller & A. Weber: KF498116/–/–; *Primulina bipinnatifida* (W.T. Wang) Y.Z. Wang: KF498113/JX506771/JX506967; *Primulina brachytricha* (W.T. Wang & D.Y. Chen) Y.Z. Wang: KF498048/-/-; *Primulina bullata* S.N. Lu & Fang Wen: KF498071/–/–; *Primulina chizhouensis* Xin Hong, S.B. Zhou & F. Wen: KF498108/–/–; *Primulina cordata* Mich. Möller & A. Weber: KF498137/–/–; *Primulina cordifolia* (D. Fang & W.T. Wang) Y.Z. Wang: KF498134/JX506776/JX506974; *Primulina danxiaensis* (W.B. Liao, S.S. Lin & R.J. Shen) W.B. Liao & K.F. Chung: JX506886/JX506780/JX506979; *Primulina depressa* (Hook. f.) Mich. Möller & A. Weber: KF498149/–/–; *Primulina dryas* (Dunn) Mich. Möller & A. Weber: –/JX195746/–; *Primulina eburnea* (Hance) Y.Z. Wang: KF498126/JX506783/JX506984; *Primulina fimbrisepala* (Hand.-Mazz.) Y.Z. Wang: KF498046/JX506787/JX506990; *Primulina glandulosa* (D. Fang, L. Zeng & D.H. Qin) Y.Z. Wang: KF498138/JX506790/JX506993; *Primulina grandibracteata* (J.M. Li & Mich. Möller) Mich. Möller & A. Weber was collected from the type locality (J.M. Li 077281); *Primulina gueilinensis* (W.T. Wang) Y.Z. Wang: KF498066/JX506749/JX506996; *Primulina guihaiensis* (Y.G. Wei, B. Pan & W.X. Tang) Mich. Möller & A. Weber: KF498078/JX506793/JX506997; *Primulina hedyotidea* (Chun) Y.Z. Wang: KF498084/JX506797/JX507000; *Primulina heterotricha* (Merr.) Y.Z. Wang: KF498099/–/–; *Primulina hezhouensis* (W.H. Wu & W.B. Xu) W.B. Xu & K.F. Chung: JX506906/JX506798/JX507003; *Primulina hochiensis* (C.C. Huang & X.X. Chen) Mich. Möller & A. Weber: KF498068/JX506796/JX507002; *Primulina huaijiensis* Z.L. Ning & J. Wang: KF498127/–/–; *Primulina jingxiensis* (Yan Liu, W.B. Xu & H.S. Gao) W.B. Xu & K.F. Chung: JX506907/JX506799/JX507004; *Primulina juliae* (Hance) Mich. Möller & A. Weber: KF498107/–/–; *Primulina langshanica* (W.T. Wang) Y.Z. Wang: KF498109/–/–; *Primulina latinervis* (W.T. Wang) Mich. Möller & A. Weber: KF498148/–/–; *Primulina laxiflora* (W.T. Wang) Y.Z. Wang: KF498079/JX506802/JX507007; *Primulina leiophylla* (W.T. Wang) Y.Z. Wang: KF498072/–/–; *Primulina leprosa* (Yan Liu & W.B. Xu) W.B. Xu & K.F. Chung: JX506861/JX506768/JX507009; *Primulina liboensi*s (W.T. Wang & D.Y. Chen) Mich. Möller & A. Weber: KF498073/JX506803/JX507010; *Primulina liguliformis* (W.T. Wang) Mich. Möller & A. Weber: JX506912/JX506804/JX507011; *Primulina lijiangensis* (B. Pan & W.B. Xu) W.B. Xu & K.F. Chung: KF498112/–/–; *Primulina linearifolia* (W.T. Wang) Y.Z. Wang: JX506913/JX506805/JX507012; *Primulina lingchuanensis* (Yan Liu & Y.G. Wei) Mich. Möller & A. Weber: JX506914/JX506806/JX507014; *Primulina lobulata* (W.T. Wang) Mich. Möller & A. Weber: KF498054/JX506807/JX507015; *Primulina longgangensis* (W.T. Wang) Y.Z. Wang: JX506863/KC765116/JX507018; *Primulina longicalyx* (J.M. Li & Y.Z. Wang) Mich. Möller & A. Weber: KF498131/–/–; *Primulina longii* (Z.Y. Li) Y.Z. Wang: KF498092/JX506809/JX507019; *Primulina longzhouensis* (B. Pan & W.H. Wu) W.B. Xu & K.F. Chung: –/JX506811/JX507021; *Primulina lunglinensis* (W.T. Wang) Mich. Möller & A. Weber: KF498097/–/–; *Primulina lungzhouensis* (W.T. Wang) Mich. Möller & A. Weber: KF498074/–/–; *Primulina luochengensis* (Yan Liu & W.B. Xu) Mich. Möller & A. Weber: KF498077/JX506812/JX507022; *Primulina lutea* (Yan Liu & Y.G. Wei) Mich. Möller & A. Weber: JX506921/JX506813/JX507023; *Primulina mabaensis* K.F. Chung & W.B. Xu: JX509622/JX506814/JX507060; *Primulina macrodonta* (D. Fang & D.H. Qin) Mich. Möller & A. Weber: KF498065/JX506816/JX507026; *Primulina medica* (D. Fang ex W.T. Wang) Y.Z. Wang: KF498094/JX506753/JX507027; *Primulina minutimaculata* (D. Fang & W.T. Wang) Y.Z. Wang: JX506865/JX506818/JX507030; *Primulina mollifolia* (D. Fang & W.T. Wang) Y.Z. Wang: JX506866/DQ872802/JX507031; *Primulina multifida* B. Pan & K.F. Chung: JQ713838/JX506819/JX507032; *Primulina nandanensis* (S.X. Huang, Y.G. Wei & W.H. Luo) Mich. Möller & A. Weber: KF498069/JX506820/JX507034; *Primulina napoensis* (Z.Y. Li) Mich. Möller & A. Weber: JX506930/JX506821/JX507035; *Primulina ningmingensis* (Yan Liu & W.H. Wu) W.B. Xu & K.F. Chung: JX506931/JX506822/JX507036; *Primulina obtusidentata* (W.T. Wang) Mich. Möller & A. Weber: KF498096/–/–; *Primulina ophiopogoides* (D. Fang & W.T. Wang) Y.Z. Wang: KF498062/–/–; *Primulina orthandra* (W.T. Wang) Mich. Möller & A. Weber: KF498147/–/–; *Primulina parvifolia* (W.T. Wang) Y.Z. Wang: KF498057/–/–; *Primulina pinnata* (W.T. Wang) Y.Z. Wang: JX506867/JX506757/JX507037; *Primulina pinnatifida* (Hand.-Mazz.) Y.Z. Wang: JX506868/JX506758/JX507038; *Primulina polycephala* (Chun) Mich. Möller & A. Weber: JX506932/JX506823/JX507040; *Primulina pseudoeburnea* (D. Fang & W.T. Wang) Mich. Möller & A. Weber: KF498060/–/–; *Primulina pseudoheterotricha* (T.J. Zhou, B. Pan & W.B. Xu) Mich. Möller & A. Weber: JX506933/JX506824/JX507041; *Primulina pseudomollifolia* W.B. Xu & Yan Liu: JX506869/JX506825/JX507042; *Primulina pteropoda* (W.T. Wang) Y.Z. Wang: KF498100/–/–; *Primulina pungentisepala* (W.T. Wang) Mich. Möller & A. Weber: JX506871/JX506828/JX507047; *Primulina purpurea* Fang Wen, Bo Zhao & Y.G. Wei: KC185416/KC185417/–; *Primulina qingyuanensis* Z.L. Ning & M. Kang: KF498129/–/–; *Primulina renifolia* (D. Fang & D.H. Qin) Y.Z. Wang: JX506737/JX506851/JX507048; *Primulina repanda* (W.T. Wang) Y.Z. Wang: KF498089/JX506832/JX507055; *Primulina ronganensis* (D. Fang & Y.G. Wei) Mich. Möller & A. Weber: JX506942/JX506833/JX507056; *Primulina rongshuiensis* (Yan Liu & Y.S. Huang) W.B. Xu & K.F. Chung: KF498088/–/–; *Primulina roseoalba* (W.T. Wang) Mich. Möller & A. Weber: KF498123/–/–; *Primulina sclerophylla* (W.T. Wang) Y.Z. Wang: JX506943/JX506834/JX507057; *Primulina shouchengensis* (Z.Y. Li) Y.Z. Wang: KF498114/JX506835/–; *Primulina spadiciformis* (W.T. Wang) Mich. Möller & A. Weber: FJ501346/–/–; *Primulina spinulosa* (D. Fang & W.T. Wang) Y.Z. Wang: JX506948/JX506839/JX507062; *Primulina subrhomboidea* (W.T. Wang) Y.Z. Wang: KF498044/JX506840/JX507063; *Primulina subulata* (W.T. Wang) Mich. Möller & A. Weber: KF498056/–/–; *Primulina swinglei* (Merr.) Mich. Möller & A. Weber: JX506950/JX506841/JX507065; *Primulina tabacum* Hance: JX506875/–/JX507066; *Primulina tenuifolia* (W.T. Wang) Y.Z. Wang: KF498058/–/–; *Primulina tenuituba* (W.T. Wang) Y.Z. Wang: KF498095/–/–; *Primulina tiandengensis* (F. Wen & H. Tang) F. Wen & K.F. Chung: KF498136/–/–; *Primulina tribracteata* (W.T. Wang) Mich. Möller & A. Weber: KF498064/JX506843/JX507067; *Primulina varicolor* (D. Fang & D.H. Qin) Y.Z. Wang: KF498086/–/–; *Primulina verecunda* (Chun) Mich. Möller & A. Weber: KF498143/–/–; *Primulina villosissima* (W.T. Wang) Mich. Möller & A. Weber: KF498145/–/–; *Primulina weii* Mich. Möller & A. Weber: DQ872832/DQ872811/–; *Primulina wentsaii* (D. Fang & L. Zeng) Y.Z. Wang: JX506953/JX506844/JX507069; *Primulina xiuningensis* (X.L. Liu & X.H. Guo) Mich. Möller & A. Weber: KF498124/JX506845/JX507074; *Primulina xiziae* Fang Wen, Yue Wang & G.J. Hua: KF498122/–/–; *Primulina yangchunensis* Y.L. Zheng & Y.F. Deng: KF498146/–/–; *Primulina yangshuoensis* Y.G. Wei & Fang Wen: –/JX506846/JX507070; *Primulina yangshanensis* W.B. Xu & B. Pan: JX506874/JX506765/JX507060; *Primulina yungfuensis* (W.T. Wang) Mich. Möller & A. Weber: KF498144/–/JX507072.

## References

[CR1] Christie F, Barber S, Möller M (2012). New chromosome counts in Old World Gesneriaceae: numbers for species hitherto regarded as *Chirita*, and their systematic and evolutionary significance. Edinb J Bot.

[CR2] Chung K-F, Huang H-Y, Peng C-I, Xu W-B (2013). *Primulina mabaensis* (Gesneriaceae), a new species from a limestone cave of northern Guangdong, China. Phytotaxa.

[CR3] Chung K-F, Leong W-C, Rubite RR, Repin R, Kiew R, Liu Y, Peng C-I (2014). Phylogenetic analyses of *Begonia* sect. *Coelocentrum* and allied limestone species of China shed light on the evolution of Sino-Vietnamese karst flora. Bot Stud.

[CR4] DeSalle R, Giribet G, Wheeler W (2002). Techniques molecular systemetics and evolution.

[CR5] Edgar RC (2004). MUSCLE: multiple sequence alignment with high accuracy and high throughput. Nucleic Acids Res.

[CR6] Farris JS, Källersjö M, Kluge AG, Bult C (1994). Testing significance of incongruence. Cladistics.

[CR7] Hall TA (1990). BioEdit: a user-friendly biological sequence alignment editor and analysis program for windows 95/98/NT. Nucl Acid S.

[CR9] Hou M-F, López-Pujol J, Qin H-N, Wang L-S, Liu Y (2010). Distribution pattern and conservation priorities for vascular plants in Southern China: Guangxi Province as a case study. Bot Stud.

[CR10] Huang Y-S, Xu W-B, Wu L, Liu Y (2012). *Primulina gongchengensis* (Gesneriaceae), a new species from Guangxi, China. Ann Bot Fenn.

[CR11] Kang M, Tao J, Wang J, Ren C, Qi Q, Xiang Q-Y, Huang H (2014). Adaptive and nonadaptive genome size evolution in Karst endemic flora of China. New Phytol.

[CR12] Li Z-Y, Wang Y-Z (2004). Plants of Gesneriaceae in China.

[CR13] Li M, Yu X-L, Ma Q-X (2014). *Primulina jiangyongensis* (Gesneriaceae), a new species from Southern Hunan, China. Phytotaxa.

[CR14] Li Q-K, Zhang Q, Deng T, Pan B, Huang Y-S, Li W-L (2015). *Primulina bobaiensis*, a new species of Gesneriaceae from Guangxi, China and its phylogenetic placement revealed by the chloroplast *matK* gene. Guihaia.

[CR15] López-Pujol J, Zhang F-M, Sun H-Q, Ying T-S, Ge S (2011). Centres of plant endemism in China: places for survival or for speciation?. J Biogeogr.

[CR16] Ning Z-L, Pan B, Kang M (2015). *Primulina fengkaiensis* (Gesneriaceae), a new species from limestone areas in western Guangdong, China. Phytotaxa.

[CR17] Pan B, Wen F, Zhao B, Deng T, Xu W-B, Huang S-X (2013). *Primulina beiliuensis* B. Pan & S. X. Huang, a new species of Gesneriaceae from limeston areas in Guangxi. China. Guihaia.

[CR18] Posada D (2008). jModelTest: phylogenetic model averaging. Mol Biol Evol.

[CR19] Ronquist F, Huelsenbeck JP (2003). MrBayes 3: Bayesian phylogenetic inference under mixed models. Bioinformatics.

[CR20] Stamatakis A, Hoover P, Rougemont J (2008). A rapid bootstrap algorithm for the RAxML web servers. Syst Biol.

[CR21] Wang W-T, Pan K-Y, Li Z-Y, Wang W-T (1990). Gesneriaceae. Flora Reipublicae Popularis Sinicae.

[CR22] Wang W-T, Pan K-Y, Li Z-Y, Weitzman AL, Skog LE, Wu Z-Y, Raven PH (1998). Gesneriaceae. Flora of China.

[CR23] Wang Y-Z, Mao R-B, Liu Y, Li J-M, Dong Y, Li Z-Y, Smith J-F (2011). Phylogenetic reconstruction of *Chirita* and allies (Gesneriaceae) with taxonomic treatments. J Syst Evol.

[CR24] Weber A, Middleton DJ, Forrest A, Kiew R, Lim C-L, Rafidah AR, Sontag S, Triboun P, Wei Y-G, Yao TL, Möller M (2011). Molecular systematics and remodelling of *Chirita* and associated genera (Gesneriaceae). Taxon.

[CR25] Wei Y-G (2010). Gesneriaceae of South China.

[CR26] Wu W-H, Meng T, Xu W-B, Liu S-Y, Zhang Q (2012). *Primulina sinovietnamica* (Gesneriaceae), a new species identified by both morphological and molecular characters from the limestone area in Guangxi, China. Phytotaxa.

[CR27] Xu Z-R (1995). A study of the vegetation and floristic affinity of the limestone forests in southern and southwestern China. Ann Mo Bot Gard.

[CR28] Xu W-B, Pan B, Liu Y, Peng C-I, Chung K-F (2012). Two new species, *Primulina multifida* and *P. pseudomollifolia* (Gesneriaceae), from karst caves in Guangxi, China. Bot Stud.

[CR29] Xu W-B, Zhang Q, Wen F, Liao W-B, Pan B, Chang H, Chung K-F (2012). Nine new combinations and one new name of *Primulina* (Gesneriaceae) from South China. Phytotaxa.

[CR30] Xu W-B, Liu Y, Kono Y, Chang H, Peng C-I, Chung K-F (2013). *Primulina cardaminifolia* (Gesneriaceae), a rare new species from limestone areas in Guangxi, China. Bot Stud.

[CR31] Xu W-B, Meng T, Zhang Q, Wu W-H, Liu Y, Chung K-F (2014). *Petrocodon* (Gesneriaceae) in the limestone karsts of Guangxi, China: three new species and a new combination based on morphological and molecular evidence. Syst Bot.

[CR32] Ying T-S, Zhang Y-L, Boufford DE (1993). The endemic genera of seed plants of China.

[CR33] Zhao B, Pan B, Zhang Y, Wen F (2013). *Primulina guizhongensis* (Gesneriaceae), a new species from Guangxi, China. Phytotaxa.

[CR34] Zheng Y-L, Deng Y-F (2014). A new species of *Primulina* (Gesneriaceae) from Guangdong, China. Phytotaxa.

[CR35] Zheng Y-L, Xia N-H, Wu T-L (2005). Gesneriaceae. Flora of Guangdong.

